# Impact of Implant Diameter on the Early Survival Rate of Dental Implants in the Saudi Population: A One-Year Retrospective Study

**DOI:** 10.7759/cureus.37765

**Published:** 2023-04-18

**Authors:** Eihab A Mously

**Affiliations:** 1 Preventive Dental Sciences, College of Dentistry Taibah University, Madina, SAU

**Keywords:** implant size, early implant success rate, early implant survival rate, implant diameter, dental implant

## Abstract

Introduction

The use of dental implants provides a revolutionary solution to the problem of missing teeth in the oral cavity. The aim of this study was to assess the early implant survival rate in relation to implant diameter and site of placement.

Methods

The data were collected from 186 patients treated between January 2019 and June 2021. All the implants were evaluated and restored after three months of implant placement. The early implant survival was calculated for different implant diameters with the odds ratio (OR).

Results

A total of 373 implants were placed. Implants were placed in the following areas: upper posterior area (UPA), n = 123, upper anterior area (UAA), n = 49, lower posterior area (LPA), n = 184, and lower anterior area (LAA), n = 17. Implants of the following diameters were placed: 3.5 mm (n = 129), 4.3 mm (n = 166), and 5 mm (n = 78). The overall early survival rate was 97.32% after three months of placement. The highest early survival rate was at LAA (100%) and the lowest early survival rate was at UAA (95.9%). The implants 5 mm in diameter had the highest early survival rate (98.72%), while the implants 3.5 mm in diameter had the lowest early survival rate (94.57%). The ORs of the early implant survival were 4.7 [95% confidence interval (CI): 0.96-23.05)] and 4.42 (95% CI: 0.53-36.61) for the 4.3 mm and 5 mm implants, respectively, with no statistical significance.

Conclusions

The implants placed in the oral cavity had acceptable survival rates regardless of implant diameter or site of placement.

## Introduction

The revolutionary invention of dental implants has provided an excellent treatment option for replacing missing teeth. With the high demand for improved dental function and aesthetics, the use of dental implants to replace missing teeth has increased phenomenally in recent decades. Implant osseointegration was first described by Brånemark et al., in 1969, as bone-to-implant contact on a microscopic level [1]. It was then defined by Alberktsson et al. as “a direct function and structural connection between living bone and the surface of a load-carrying implant.” [2]. With improvements in implant surface and design, it is widely accepted that the success rate of implant osseointegration has increased to around 95%, ranging from 93% to 98% [3-5]. Implant failure can be divided by the time of failure into early and late implant failure [6]. An early implant failure results from the failure of the bone to establish “an intimate bone-to-implant contact.” [7]. Late implant failure occurs after implant loading. In a literature review studying the survival rate of 33,922 implants, six studies reported that the survival rate was not influenced by implant diameter. Five studies reported a survival rate of implants 4.5 mm or more in diameter that ranged from 91.8% to 100%. Seven studies reported survival rates of narrow-diameter implants with a mean of 95.5% and a range of 93.3 to 99.4%) [8]. In a study of 1649 implants placed, the highest failure rate was reported for narrow-diameter implants, which was 5.1%, compared to 3.8% for regular-diameter implants and 2.7% for wide-diameter implants [9]. In a review of 10,093 small-diameter implants (implants ≤ 3.5 mm) across 41 studies, the survival rates of the implants were all above 90% [10]. In a meta-analysis of 3291 implants placed, a statistically significant difference was found in the failure rate of implants with diameters < 3.3 mm (1.21%) compared to implants with diameters ≥ 3.3 mm (0.34%) [11]. In a recent study of 2053 implants placed in 1078 patients, the implant survival rates for implants < 3.75 mm, 3.75-5 mm, and ≥ 5 mm were 95.2%, 96.54%, and 94.96%, respectively. Furthermore, there is no consensus or universal classification that determines which implant diameter should be considered a narrow-diameter implant. One study considered any implant with a diameter ≤ 3.7 mm as a narrow-diameter implant. As for implants of a 3.8 mm diameter and more, they would be considered standard diameter implants [12]. Another study defined implants ≤ 3 mm, in diameter as narrow-diameter implants [13]. Another report considered implants ranging from 3.0 to 3.4 mm in diameter to be narrow-diameter implants. Moreover, Quek et al. proposed to classify implants into wide (5 to 6 mm), regular (3.75 to 4 mm), narrow or small (3 to 3.4 mm), and mini (< 2.9 mm) [14]. This resulted in huge variability in the definition of narrow-diameter implants.

At present, there are limited data on the prevalence of osseointegration and early implant failure in the Saudi population. In a three-year retrospective study conducted in Riyadh, Saudi Arabia, on immediate implant placement to replace single-rooted teeth, the early survival rate was 96% [15]. The aim of this study was to assess the early implant survival rate in relation to implant diameter and placement site.

## Materials and methods

This was a retrospective cohort study conducted at a private practice in Jeddah, Saudi Arabia. The patients were referred to the implant clinic by a prosthodontic consultant for the replacement of missing teeth with dental implants. The sample size was limited to patients treated from January 2019 to June 2021. Exclusion criteria included uncontrolled systemic diseases such as diabetes, hypertension, and other uncontrolled systemic conditions. Written consent forms were obtained from all patients undergoing the surgical procedures, implant placement, and data use for research purposes. The data for all the patients were de-identified to protect the confidentiality of the patients. The study was ethically approved by the Taibah University College of Dentistry Research Ethical Committee (TUCDREC), with reference number EAMously/TUCDREC/021222.

Treatment

**Figure 1:**
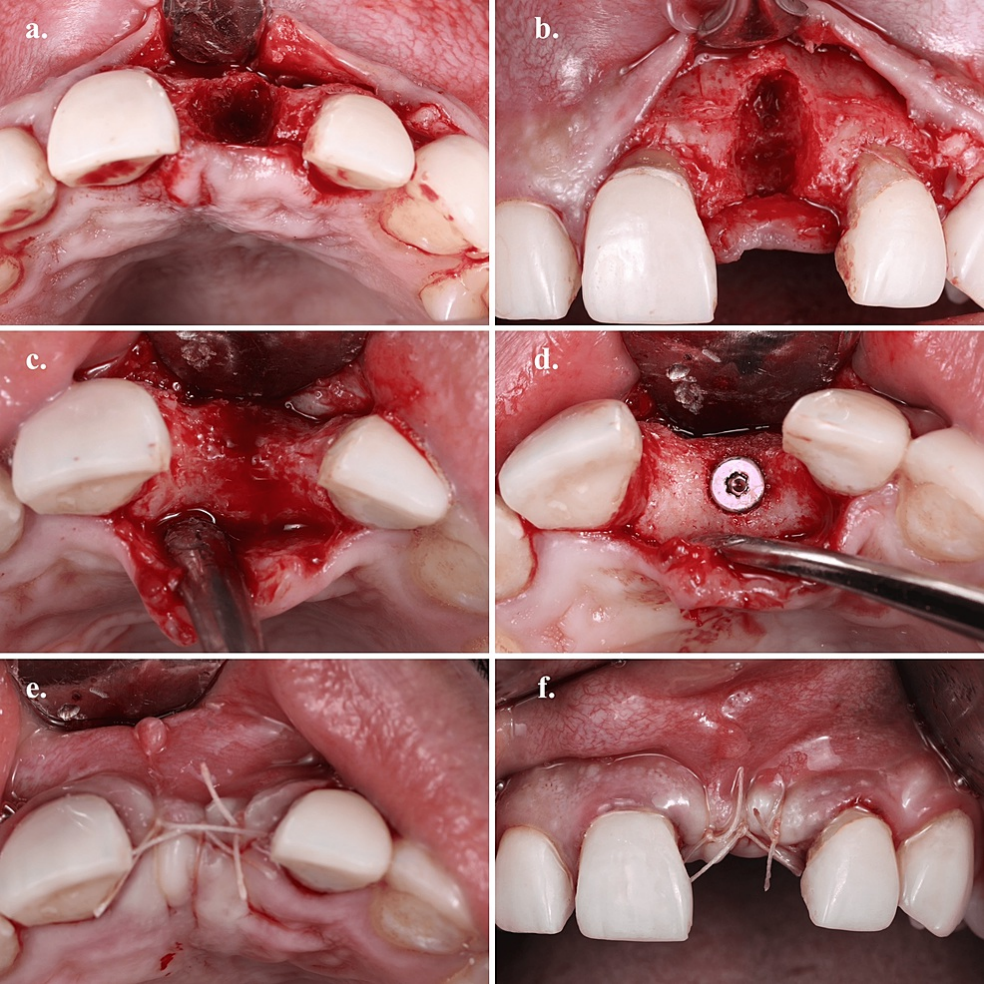
Dental implant placement. (a) Occlusal view after flap reflection. (b) Frontal view after flap reflection showing the amount of bone loss. (c) Occlusal view of the alveolar ridge after 3 months of bone graft. (d) 3.5x10 mm implant placement. (e) Occlusal view after suturing. (f) Frontal view after suturing.

All the implants (Noble Replace Select Tapered; Noble BioCare, Gutenberg, Sweden) were placed by a single operator (Figure 1). The operator was a US-trained consultant and an American-board-certified periodontist. The same preoperative, intraoperative, and postoperative protocols were followed with all patients. After assessing a cone beam computed tomography (CBCT), bone intervention and grafting techniques were one of the following: either a socket preservation graft using mineralized allograft bone particles (FDBA, Mineross, Biohorizons IPH, Birmingham, AZ, USA), guided bone regeneration (GBR) with mineralized allograft bone particles (FDBA, Mineross, Biohorizons IPH, Birmingham, AZ, USA) and a resorbable collagen membrane (Mem-Lok, Biohorizons IPH, Birmingham, AZ, USA), or external sinus lift technique using the same materials as needed. The healing period for the grafted areas was one of the following: three to four months for socket preservation techniques cases, three to four months for small GBR cases, and six months for large GBR and external sinus lift cases. The decision on the healing period between small and large GBR sites was solely dependent on the clinical judgment of the operator. Following the International Team for Implantology (ITI) latest guidelines published in 2018 [16], all implant placement and loading protocols were early implant placement with conventional implant loading (Type 3C) except for large GBR and sinus lift cases which was late implant placement with conventional implant loading (Type 4C). A flapped two-stage approach was used to place the implants. The implant diameter was determined by carefully assessing the bone thickness using a CBCT. All implants were placed with an insertion torque of 35 Ncm or above and the lengths of all implants were 10 mm or above which is considered standard implant length [17]. 

Data

All the patients were evaluated one week after the implant placement surgery, three months after surgery during the second stage, and one year after prosthetic restoration placement. The implants were examined clinically and radiographically. Clinical evaluation of implant osseointegration by direct vision and reverse torquing of 35 Ncm, absence of pain, and peri-implant mucositis and peri-implantitis were noted. Radiographic evaluations, including periapical radiographs, were conducted to assess osseointegration and changes in crestal bone levels.

Statistical analysis

A chi-square test was conducted to assess if the failure of the implants was dependent on age, gender, and grafting status. The implants were subcategorized based on the diameter and the site of placement. Next, the early implant survival was calculated for each category. Furthermore, the odds ratios (ORs) of the early survival of the implants and their diameter were calculated. The p-value was set at the level of significance at 0.05. All statistical analyses were performed using Stata/MP V.15 (StataCorp, College Station, TX, USA).

## Results

From January 2019 to June 2021, a total of 186 patients (52 males (27.96%), and 134 females (72.04%); ages ranged from 21-75 years with a mean age of 43.1) met the inclusion criteria for implant placement. A total of 373 implants were placed. As shown in Table 1, the age of the patient was equally distributed among the sample with 61 (32.8 %) implants placed in patients less than 35 years old, 50 (26.88%) implants placed in patients of 35 to 44 years old, 33 (17.74%) implants placed in patients of 45 to 54 years old, 30 (16.13%) implants placed in patients of 55 to 64 years old, and 12 (6.45%) implants placed in patients more than 65 years old. 188 (50.4%) of the implant placed in non-grafted sites while 185 (49.6%) implants were placed in grafted sites. The number of implants that failed in female patients was 8/10 (80%) implants compared to 2/10 (20%) implants failed in male patients with no statistical significance between gender groups. Regarding implant failure and age groups, 3/10 (30%) of implants failed in patients less than 35 years old. 5/10 (50%) implants failed in patients 35 to 44 years old group, and 2/10 (20%) implants failed in patients 45 to 54 years old with no statistical significance in all age groups. Regarding grafting status, 7/10 (70%) implant failure occurred in non-grafted sites compared to 3/10 (30%) implant failure in grafted sites with no statistical significance.

**Table 1:** Demographic characteristics and graft status of the patients by the implants failed ^a ^n= number of cases, * Chi-square test was performed. The p-value was set at a .05 level of significance. All the p-values were not significant.

Gender	n^a^ (%)	Failure n (%)	P-value*
Male	52 (27.96%)	2 (20%)	0.550
Female	134 (72.04%)	8 (80%)	
Age			
Mean (43.1)			
less than 35	61 (32.80%)	3 (30%)	0.488
35 to 44 years old	50 (26.88%)	5 (50%)	
45 to 54 years old	33 (17.74%)	2 (20%)	
55 to 64 years old	30 (16.13%)	-	
more than 65	12 (6.45%)	-	
Graft status			
Non-grafted site	188 (50.4%)	7 (70%)	0.209
Grafted site	185 (49.60%)	3 (30%)	

As shown in Table 2, the site of implant placement was divided as follows: upper posterior area (UPA) n = 122, upper anterior area (UAA) n = 47, lower posterior area (LPA) n = 177, and lower anterior area (LAA) n = 17. The early implant survival rate was 97.32% (n = 363). The early implant failure rate was 2.68% (n = 10).

**Table 2:** Implant survival rates based on site of implant placement. *bone type according to (Lekhom and Zarb [18] classification)

Site of Implant Placement*	Implants Placed (n)	Implants Survived		Implants Failed	
		n	%	n	%
upper posterior area (UPA) D4 bone type	123	122	99.19	1	0.81
upper anterior area (UAA) D3 bone type	49	47	95.9	2	4.08
lower posterior area (LPA) D2 bone type	184	177	96.20	7	3.80
lower anterior area (LAA) D1 Bone type	17	17	100%	-	-
Total	373	363	97.32	10	2.68

The implants placed in the UAA had the lowest survival rate, of 95.9% (2/49 implants failed). The LPA had the highest number of failures (7/177 implants failed), with a survival rate of 96.2%. In the UPA, only one implant failed (1/123 implants failed), with a survival rate of 99.19%. In the LAA, no implant failure was present, with a survival rate of 100%. All patients who demonstrated implant failure were non-smoker patients.

As described in Table 3, the implant diameter of 3.5 mm had the lowest survival rate, of 94.57% (7/129 implants failed). The survival rates of the 4.3 mm (2/166 implant failed) and 5 mm (1/78 implant failed) implants were very similar at 98.8% and 98.72%, respectively.

**Table 3:** Implant survival rates based on diameter of implant placed.

Diameter of Implant Placed (mm)	Implants Placed (n)	Implants Survived		Implants Failed	
		n	%	n	%
3.5 mm	129	122	94.57	7	5.43
4.3 mm	166	164	98.80	2	1.20
5 mm	78	77	98.72	1	1.28
Total	373	363	97.32	10	2.68

When combining the implant placement site with the implant diameter, the lowest survival rate was 90% (4/40 implants failed), for the 3.5 mm implants placed in the LPA (Table 3), followed by the 3.5 mm diameter implants (2/40 implants failed) placed in the UAA (95%). For the 3.5 mm implants placed in the UPA, the survival rate was 97.06% (1/34 implants failed). Two 4.5 mm implants placed in the LPA failed (2/97 implants failed), with a survival rate of 97.94%, and one 5 mm implant failed when placed in the LPA (1/47 implants failed), with a survival rate of 97.87%. The remaining implants of various diameters placed in other sites showed no failure (Table 4).

**Table 4:** Sizes of implants placed at different extraction sites and their survival rates. *bone type according to (Lekhom and Zarb [18] classification)

Size of Implant Placed (mm)	3.5 mm			4.5 mm			5 mm		
	Implants Placed (n)	Implants Failed (n)	Implants Survived %	Implants Placed (n)	Implants Failed (n)	Implants Survived %	Implants Placed (n)	Implants Failed (n)	Implants Survived %
upper posterior, D4 bone type*	34	1	97.06	58	0	100	31	0	100
upper anterior, D3 bone type*	40	2	95	9	0	100	-	-	-
lower posterior, D2 bone type*	40	4	90	97	2	97.94	47	1	97.87
lower anterior, D1 bone type*	15	0	100	2	0	100	-	-	-

The ORs of implant survival were 4.7 (95% CI: 0.96-23.05) and 4.42 (95% CI: 0.53-36.61) for the 4.3 mm and 5 mm implants, respectively. However, the results were not statistically significant (Table 5).

**Table 5:** Odds ratios of survival based on implant diameter.

Implant Diameter	Reference Group	OR	95% CI	p Value
4.3 mm	3.5 mm	4.70	(0.96–23.05)	0.056
5 mm	3.5 mm	4.42	(0.53–36.61)	0.169

## Discussion

With technological advancements in implant design and surface treatment, an improvement in survival rate is expected. The implant size, including diameter and length, is determined by the thickness and height of the alveolar ridge. In this study, the overall early survival rate was 97.32%. The implants 3.5 mm in diameter showed the lowest survival rate, of 94.57%, followed by those measuring 4.3 mm and 5 mm, which showed comparable survival rates at 98.8% and 98.72%, respectively. Although statistically insignificant, the ORs of the survival of the 4.3 mm and 5 mm implants were 4.7 (95% CI: 0.96-23.05) and 4.42 (95% CI: 0.53-36.61), respectively. All implant failures occurred during the three months implant second-stage visit.

This is consistent with the results presented by Olate et al. [9]. They found that the failure rates of narrow-, regular- and wide-diameter implants were 5.1%, 3.8%, and 2.7%, respectively. This was also consistent with a meta-analysis that found that narrow implants have statistically significant lower survival rates, although they stated that the exact time of failure was not specified in most of their screened articles, which caused the authors to assume that the implant failure occurred at the end of the observational period [11].

In an earlier review by Renouard and Nisand in 2006 (study period 1990-2005), the data showed that most articles reported higher failure rates with wide-diameter implants, except for one article, which reported a higher failure rate with narrow-diameter implants [8]. In another review of small-diameter implants, all the reports demonstrated survival rates of 90% and above [10]. In a meta-analysis conducted in 2014, the failure rate ranged from 0 to 4.12% [11]. This might be explained by developments and improvements in implant design and surface treatment. Increased bone-to-implant contact (BIC) is evident in wider-diameter implants, which might contribute to the increased survival rates of wide-diameter implants in the newer models.

The strength of this study lies in the fact that all the implants were placed by a single well-trained operator following the same surgical protocol and decision-making process when the implant placement was planned. One of the limitations of this study was that we did not assess various types of implants. The inclusion of low-cost implants with less evidence of success might influence the early survival rate. Another limitation related to this cohort is that selection bias cannot be eliminated as the sample used in the study was a convenience sample.

In the articles mentioned above, the cut-offs between narrow-diameter implants and standard-diameter implants varied, but all the implant diameters were less than the range of 3.3 mm to 4 mm. Currently, there is no standard definition of narrow-diameter implants. This might provide confusing and contradictory results. Thus, more studies need to be conducted to determine the exact diameter that should be considered a narrow-diameter implant to maximize the successful outcomes of implant surgeries. Hence, future studies should analyze a broader range of implant diameters in order to obtain more comprehensive results Moreover, more controlled studies need to be performed to pinpoint the non-patient-related causes of implant failure.

## Conclusions

To the limitations of this study, there were no significant differences in the early survival rates of implants regarding implant diameter and site of placement. However, narrow diameter implants demonstrated an increased risk of implant failure. More randomized controlled studies need to be conducted to determine the cut-off of narrow-diameter implants and its relation to implant failure. Therefore, future studies should analyze a broader range of implant diameters in order to obtain more comprehensive results.
